# Effect of Ultrasound-Guided Sciatic and Saphenous Nerve Blocks on Analgesia During General Anaesthesia for Ankle Fracture Surgery: A Randomised Controlled Trial

**DOI:** 10.3390/medicina62071410

**Published:** 2026-07-21

**Authors:** Hyun Ji John, Jong Bum Choi, Min Soo Jang, Chi Young Lee, Seungwon Jeong, Jong Yeop Kim, Mazen Zein, Jae Yong Park, Soo Kyung Lee, Yi Hwa Choi

**Affiliations:** 1 Department of Anaesthesiology and Pain Medicine, College of Medicine, Hallym University, Hallym University Sacred Heart Hospital, Anyang 14068, Republic of Korea; jhj22@hallym.or.kr (H.J.J.); 210315@hallym.or.kr (M.S.J.); leecy1782@hallym.or.kr (C.Y.L.); mim1343@hallym.or.kr (S.J.); agneta@hallym.or.kr (S.K.L.); 2 Department of Anaesthesiology and Pain Medicine, College of Medicine, Ajou University School of Medicine, Suwon 16499, Republic of Korea; romeojb@naver.com (J.B.C.); kjyeop@ajou.ac.kr (J.Y.K.); 3 Interventional Spine & Pain Medicine, Department of Neurosurgery, Division of Spine Medicine, Duke University School of Medicine, Durham, NC 27710, USA; mazen.zein@duke.edu; 4 Department of Orthopaedic Surgery, College of Medicine, Hallym University, Hallym University Sacred Heart Hospital, Anyang 14068, Republic of Korea; getfours@hallym.or.kr

**Keywords:** analgesics, anaesthesia, nerve block, postoperative pain, regional analgesia, ultrasound guidance

## Abstract

*Background and Objectives*: Ultrasound-guided sciatic and saphenous nerve blocks are widely used as analgesic alternatives for multimodal analgesia. However, how to objectively quantify the opioid- and anaesthetic-sparing effects of an additional peripheral nerve block in patients undergoing ankle fracture surgery under general anaesthesia remains unclear. This study aimed to evaluate their efficacy in managing postoperative pain and opioid-sparing effects following ankle fracture surgery. *Materials and Methods*: Patients scheduled for ankle fracture surgery were randomly assigned to the nerve block (*n* = 30) or control group (*n* = 30). The primary outcome was the postoperative numeric rating scale (NRS) score. The secondary outcomes included the total doses of remifentanil and propofol, Quality of Recovery-40 Korean (QoR-40K) scores, and self-reported patient satisfaction. *Results*: The NRS score immediately after surgery was significantly lower in the nerve block group compared with that in the control group (2.6 ± 2.5 vs. 6.2 ± 2.6; *p* < 0.001). The total remifentanil dose required during surgery was also significantly lower in the nerve block group than in the control group (1148.1 ± 391.8 vs. 1487.4 ± 798.8; *p* = 0.041). However, the patient satisfaction scale (3 [IQR, 2–4] vs. 3 [IQR, 2–4]; *p* = 0.206) and QoR-40K score 24 h after surgery (169 [IQR, 155–184] vs. 175 [IQR, 154–165]; *p* = 0.324) did not differ significantly between the two groups. *Conclusions*: Ultrasound-guided sciatic and saphenous nerve blocks significantly reduced immediate postoperative pain and intraoperative remifentanil consumption under qNOX (quantitative nociception index)-guided general anaesthesia during ankle fracture surgery.

## 1. Introduction

Excessive nociceptive stimulation during surgery affects the length of a hospital stay, overall hospital care costs, and patient outcomes [1,2]. The intensity of postoperative pain immediately after surgery correlates with the risk of developing long-term postsurgical pain [3]. Optimal interventions for postoperative pain control are important to improve postoperative recovery and surgical outcomes as well as to attenuate the development of chronic pain [4].

Perioperative and postoperative analgesics are usually opioid-based. Remifentanil, an opioid widely used for general anaesthesia owing to its short-acting properties, is associated with acute opioid tolerance [5]. Intraoperative opioid overdose caused by inadequate analgesia may lead to side effects such as acute opioid tolerance and postoperative hyperalgesia [6]. These effects can increase pain intensity and duration, paradoxically leading to increased usage of postoperative analgesics and adverse outcomes, including nausea and vomiting, which significantly affect patient satisfaction following surgery.

The EEG-based hypnotic and analgesic depth monitors have become an essential part of anaesthesia in recent years. The qNOX (quantitative nociception index) sensor is compatible with the CONOX^®^ monitor (Fresenius Kabi, Bad Homburg, Germany). The qNOX index is a central nervous system-based monitoring tool used to predict responses to noxious stimulation during sedation and general anaesthesia [7]. The values are derived from electroencephalography analysis and interpreted through beta arousals, delta arousals, and alpha dropouts [8,9]. The likelihood of a movement response to noxious stimuli is indicated on a scale from 0 to 100. A value above 60 corresponds to a high probability of response to surgical noxious stimuli, whereas a value below 40 indicates a very low probability of response. In this study, the remifentanil concentration was adjusted to maintain qNOX values between 40 and 60.

Ultrasound-guided peripheral nerve blocks have been increasingly employed to manage postoperative pain. In particular, these anaesthetic techniques could improve postoperative recovery for the patients with ankle fractures who often experience severe postoperative pain. In addition, regional analgesia is used to suppress the development of chronic postoperative neuropathic pain by alleviating the preceding pain induced by the surgery [10] and thereby improving patient satisfaction after surgery. Regional analgesic techniques also prevent opioid-induced hyperalgesia and tolerance by reducing perioperative opioid use [2]. Despite peripheral nerve block being a cornerstone of multimodal analgesia for opioid-sparing/opioid-free anaesthesia, its objectively quantitative opioid-sparing effect has not been well established.

This study aimed to evaluate the efficacy of ultrasound-guided sciatic and saphenous nerve blocks on the intensity of postoperative pain measured by numeric rating scale (NRS) scores within 24 h after ankle fracture surgery under general anaesthesia. The total doses of remifentanil and propofol administered for general anaesthesia were evaluated in each group of patients to identify the potential of regional analgesia to reduce intraoperative opioid consumption based on the CONOX^®^ value [7,11], which quantifies the depth of anaesthesia and nociception index during the perioperative period. Patient satisfaction scores were also evaluated.

## 2. Materials and Methods

This prospective, randomised, patient-blinded study was conducted from May 2020 to August 2022 in accordance with the Declaration of Helsinki, following approval from the Institutional Review Board of our institution. Written informed consent was obtained from all participants prior to enrolment. The study was retrospectively registered at the Clinical Research Information Service, Korea Centres for Disease Control and Prevention, Ministry of Health and Welfare (cris.nih.go.kr), with the unique identifier: KCT0007632.

We initially screened 60 patients with American Society of Anaesthesiologists physical status I–III, aged >19 years, who were scheduled for ankle fracture surgery under general anaesthesia at our institution. The exclusion criteria were as follows: history of allergic reaction to local anaesthetics, acute infection or ongoing septic conditions, and morbid obesity (body mass index > 35 kg/m^2^). Patients were randomly allocated to the control and nerve block (NB) groups using a computer-generated random number table. The randomisation sequence was generated using a block size of four.

No premedication was administered. All patients were monitored in the operating room using electrocardiography, non-invasive arterial blood pressure measurement, pulse oximetry, capnography, and a CONOX^®^ sensor (CONOX^®^, Quantium Medical S.L.U., Mataró, Barcelona, Spain) applied to the forehead. After pre-oxygenation, propofol (effect-site concentration, 5.0 μg/mL) and remifentanil (effect-site concentration, 4.0 μg/mL) were administered through a target-controlled infusion pump (Orchestra^®^ target-controlled infusion pump, Fresenius Vial, Brezins, France) along with midazolam (0.05 mg/kg) for anaesthesia induction. Once the patient became unconscious, rocuronium (0.6 mg/kg) was injected, and manual mask ventilation was performed. After 1 min, orotracheal intubation was facilitated (tube size: 7.0 mm for females and 7.5 mm for males). During anaesthesia, propofol and remifentanil were adjusted to maintain qNOX and qCON (quantitative consciousness index) values between 40 and 60. Patients randomly allocated to the NB group received an ultrasound-guided saphenous/sciatic nerve block.

In the NB group, patients were prepared with chlorhexidine, and a sterile drape was placed over the affected leg after intubation. A linear probe (LOGIQ^TM^ P9, GE, Chicago, IL, USA) was placed perpendicular to the long axis of the medial thigh with the knee slightly flexed and the hip externally rotated in the supine position to identify the distal end of the adductor canal, where the saphenous nerve is located between the sartorius and vastus medialis. A 60 mm, 23-gauge block needle (KOVAX-needle 60 mm, Korea Vaccine, Seoul, Korea) was inserted using an in-plane approach. After confirming that the needle tip was near the saphenous nerve, a total of 10.5 mL of 0.5% ropivacaine was administered while observing the spread of the local anaesthetic. The patient was then repositioned to lie on the left or right side, with the operative side up. An ultrasound-guided popliteal sciatic nerve block was performed just above the bifurcation of the sciatic nerve, and 16 mL of 0.5% ropivacaine was injected. All nerve blocks were performed by an experienced anaesthesiologist with over 10 years of regional anaesthesia experience under ultrasound guidance. The needle position was confirmed by hydrodissection with a small amount of normal saline showing circumferential spread of the injectate around the nerve. After negative aspiration to exclude intravascular injection, the local anaesthetic was administered.

All patients in both groups were prescribed patient-controlled analgesia (PCA) with fentanyl citrate 15 mcg/kg and ramosetron 0.6 mg in normal saline (a total volume of 100 mL). The PCA pump was set at a basal infusion rate of 2 mL/h, an intermittent bolus of 0.5 mL, and a lockout interval of 15 min. The PCA in the NB group started after the recovery of sensation in the limb that had undergone a nerve block. In the control group, PCA was initiated at the time of skin incision according to our institutional protocol. To prevent postoperative nausea and vomiting, 0.3 mg of ramosetron was administered intravenously at the end of surgery.

The primary outcome was the postoperative NRS score, numerically rated from 0 to 10. In both groups, the NRS score was checked in the post-anaesthesia care unit (PACU) immediately before discharge from the recovery room, 6 h after surgery, and on postoperative day (POD) 1. The secondary outcomes were the total dose of remifentanil administered during surgery and Quality of Recovery-40 (QoR-40) after surgery [12].

Haemodynamic profiles, including mean arterial pressure, heart rate, arterial oxyhaemoglobin saturation, and qCON-qNOX values (hypnotic and analgesic monitoring), were recorded at baseline, immediately before intubation, 1 min after intubation, 10 min after intubation, during skin incision, at the end of surgery, and upon arrival in the PACU. The total dose of infused propofol, use of atropine and ephedrine, and rescue fentanyl dose were recorded. Rescue fentanyl was administered when patients complained of pain at the surgical site during the postoperative 24 h. Bradycardia < 50 min was treated with intravenous atropine (0.5 mg), and if patients showed a mean arterial pressure <60 mmHg, they were treated with ephedrine. Patient satisfaction scores were assessed on POD 1.

Categorical variables are shown as frequencies with percentages, whereas continuous variables are shown as medians and interquartile ranges or means and standard deviations. Categorical variables were compared using the chi-squared test, and continuous variables were analysed using Student’s *t*-test. Based on a previous study by Short et al. [13], the standard deviation of the NRS score (0–10) was expected to be 2.5 points. We assumed that the NRS score could show a clinically meaningful reduction of 2 points in the NB group. It was estimated that 30 patients in each group would be required for a probability of an alpha error of 5%, a study power of 80%, and an expected dropout rate of 15%. All statistical analyses were performed using SPSS (version 21.0, IBM, Chicago, IL, USA). Effects were considered statistically significant if the null hypothesis could be rejected at the 0.05 probability level.

## 3. Results

**Figure 1:**
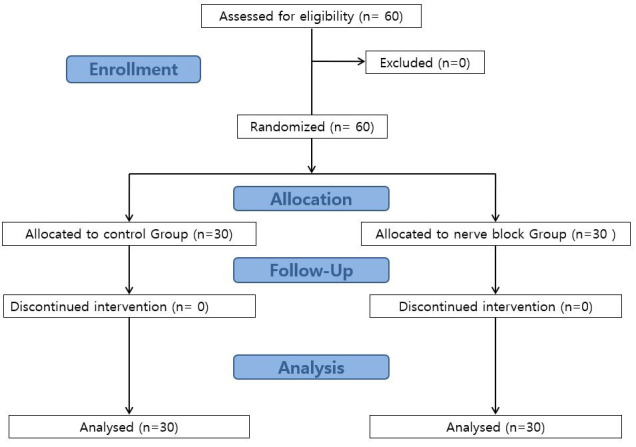
Flow diagram.

The number of patients screened, randomised, and assigned to each study group is shown in Figure 1. Between March 2020 and August 2022, a total of 60 patients were enrolled. Of these, 30 patients were randomly assigned to the NB group and 30 to the control group.

The baseline characteristics were comparable between the groups (Table 1). All nerve block procedures in the NB group were successful and free of significant complications.

The overall anaesthesia time was not significantly different between the two groups. The total remifentanil dose required for general anaesthesia was significantly lower in the NB group than in the control group (1148.1 ± 391.8 vs. 1487.4 ± 798.8, *p* = 0.041) (Table 2). The total propofol dose was comparable between the groups (1047.6 ± 391.8 vs. 1279.1 ± 629.2; *p* = 0.084).

**Figure 2:**
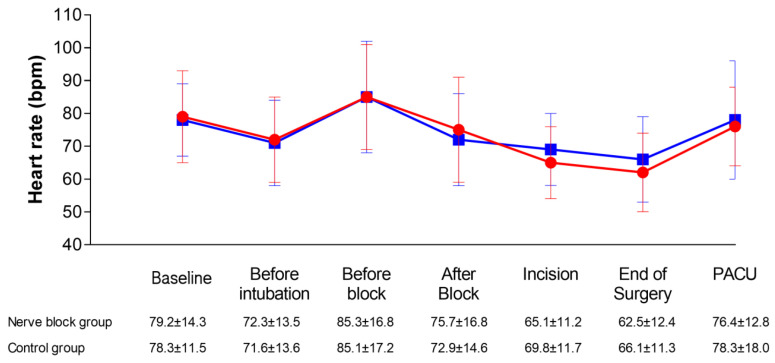
Haemodynamic changes: heart rate. Red: Nerve block. Blue: Control.

**Figure 3:**
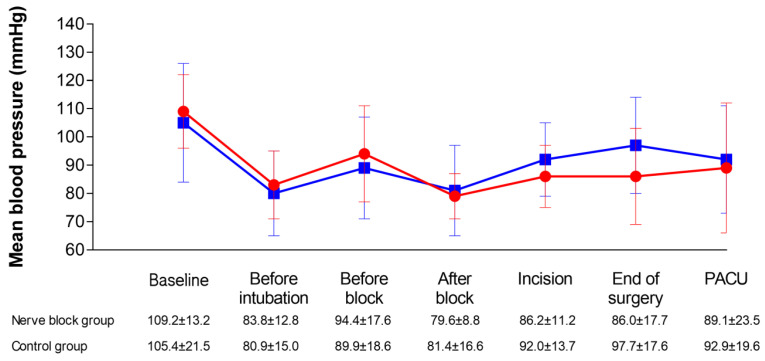
Haemodynamic changes: mean blood pressure. Red: Nerve block. Blue: Control.

**Figure 4:**
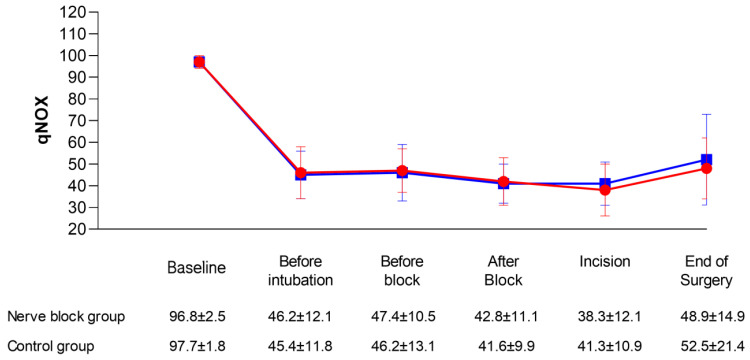
CONOX value (qNOX). Red: Nerve block. Blue: Control.

**Figure 5:**
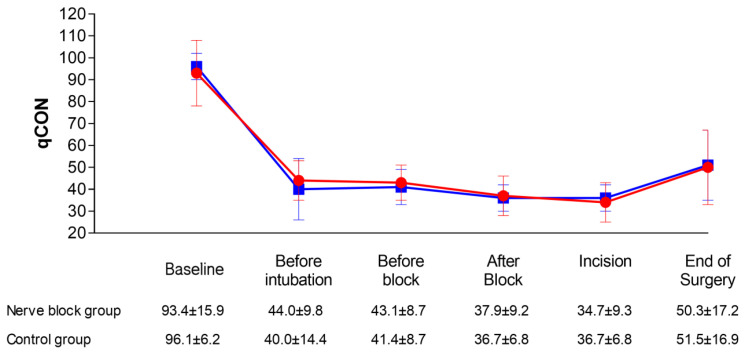
CONOX value (qCON). Red: Nerve block. Blue: Control.

Haemodynamic changes and CONOX^®^ (qNOX/qCON) values during surgery are summarised in Figure 2, Figure 3, Figure 4 and Figure 5. During surgery, heart rate and mean blood pressure decreased before intubation and then rapidly increased after intubation. Subsequently, after the block was performed, both parameters showed a sharp decline and remained stable throughout surgery. The qCON and qNON values exhibited similar trends. Chronological changes in haemodynamic monitoring measures were not significantly different between the groups (*p* = 0.841, 0.605, 0.779, and 0.688 for HR, MBP, qCON, and qNOX, respectively, based on repeated-measures generalised linear modelling). Three patients in the NB group and four in the control group were administered intravenous ephedrine, and one patient in the control group was injected with atropine.

**Figure 6:**
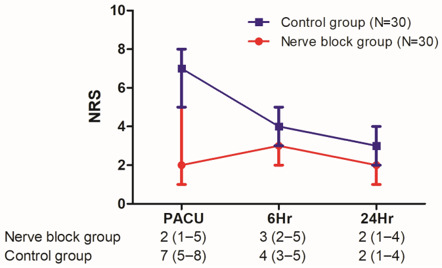
Changes in postoperative pain intensity over time.

The primary study outcome, the NRS score immediately after surgery, was significantly lower in the intervention group than in the control group (2 [IQR, 1–5] vs. 7 [IQR, 5–8]; *p* < 0.001) (Table 3). However, NRS values were comparable between the groups at 6 h (3 [IQR, 2–5] vs. 4 [IQR, 3–5]; *p* = 0.068) and 24 h after surgery (2 [IQR, 1–4] vs. 2 [IQR, 1–4]; *p* = 0.740) (Figure 6).

The patient satisfaction scale (3 [IQR, 2–4] vs. 3 [IQR, 2–4]; *p* = 0.206) and QoR-40K score 24 h after surgery (169 [IQR, 155–184] vs. 175 [IQR, 154–165]; *p* = 0.740) did not differ significantly. Rescue analgesic use was required in 16.7% and 10.0% of patients in the NB and control groups, respectively (*p* = 0.706).

## 4. Discussion

This study demonstrated that ultrasound-guided sciatic and saphenous nerve blocks significantly reduced intraoperative remifentanil requirements under qNOX-guided general anaesthesia and were associated with lower postoperative NRS scores during the immediate postoperative period in patients undergoing ankle fracture surgery. However, no significant differences were observed between the groups in patient satisfaction or QoR-40K values on postoperative day 1.

Regional analgesia is known to reduce postoperative pain, opioid use, antiemetic use, and hospital stay compared with opioids alone [14]. In addition, regional analgesic techniques provide superior same-day recovery and are effective options for early recovery after fracture surgery [15]. Among multimodal analgesia approaches, these techniques offer the most profound opioid-sparing effect during fracture surgery. Ankle fracture surgery is associated with the highest level of postoperative pain within the first 24 h [16,17]. The perioperative and postoperative period also involves the greatest analgesic requirements including opioids. The duration of this study was from surgery until postoperative 24 h. In this study, despite significantly lower immediate postoperative NRS scores at PACU in the NB group than those in the control group, this analgesic benefit did not persist. Contrary to our expectations, no statistically significant differences in NRS scores were observed at 6 (*p* = 0.068) or 24 h (*p* = 0.740) postoperatively. These findings may suggest that no additional analgesic benefit was obtained beyond the duration of action of the local anaesthetic. Also, the QoR-40 and patient satisfaction scores on POD 1 did not show any difference between groups. This might be attributable to the nature of total intravenous anaesthesia (TIVA), which is associated with a fast postoperative recovery and higher physical comfort [18]. Patients undergoing TIVA usually report satisfactory recovery, including enhanced postoperative comfort and prompt resumption of normal activities [19]. All patients in both groups were provided TIVA during this study. No group showed significantly better results in QoR-40 and patient satisfaction scores, despite lower NRS scores in the intervention group immediately after surgery consistent with previous findings.

Inadequate perioperative analgesia can lead to intraoperative opioid overuse and potential opioid-related side effects such as postoperative hyperalgesia. Opioid-induced hyperalgesia is a paradoxical increase in pain sensitivity. Its clinical features are described as increased pain and opioid consumption [20]. The mechanism of opioid-induced hyperalgesia is still unclear, but it may result from the activity of central glutamatergic systems, the release of spinal dynorphins, descending facilitation, a certain genotype, transient receptor potential cation channel subfamily V activity, or increased 5-hydroxytryptamine levels [21]. Previous studies reported that remifentanil-mediated hyperalgesia correlates with high-dose infusions and high cumulative doses during anaesthesia [22,23]. In this study, remifentanil consumption during anaesthesia was significantly lower in the NB group than in the control group. Furthermore, the immediate postoperative NRS at the PACU was higher in the control group (*p* < 0.001). A previous study reported findings contrary to ours [24], demonstrating that peripheral nerve block reduced anaesthetic requirements but not analgesic consumption. Kamiya et al. did not appear to use nociception monitoring during surgery. In contrast, our findings show that sciatic and saphenous nerve blocks reduced intraoperative remifentanil consumption without affecting the total propofol dose during qNOX-guided analgesia and qCON-guided hypnosis. Adequate perioperative peripheral nerve block may facilitate a reduction in intraoperative opioid requirements by attenuating nociceptive input, which could subsequently influence the development of opioid-induced hyperalgesia.

The CONOX^®^ (qCON-qNOX) used in this study is a non-invasive depth of anaesthesia monitor that assesses the hypnotic and analgesic effects of sedation and general anaesthesia. The qCON assesses the patients’ state of consciousness. The scale between 40 and 60 indicates adequate anaesthesia. The qNOX index is used to assess to predict responses to noxious stimulation during general anaesthesia [7]. A qNOX value between 40 and 60 signifies a low likelihood of a response to a noxious stimulus during surgery.

Concerns have been raised regarding a rebound pain associated with regional analgesic techniques in acute ankle fracture surgery [17,25]. In this study, the NRS scores in the NB group were slightly higher at 6 h after surgery than at PACU discharge (2.6 ± 2.5 vs. 3.3 ± 2.2; *p* = 0.157); however, the scores did not exceed those in the control group at 6 h, and after 24 h, the scores changed similarly in both groups. No extreme fluctuations in NRS scores were observed during the 24 h following surgery in the NB group. Rescue fentanyl to control postoperative pain was administered to five patients in the NB group and three patients in the control group, and this difference was not statistically significant. In contrast to previous studies, this study did not demonstrate clinically significant rebound pain requiring additional postoperative opioid administration.

This study had some limitations. First, the investigator was not blinded owing to the nature of the study. However, all intraoperative and postoperative outcomes were recorded by independent medical personnel who were not involved in this study to minimise subjective judgement, reducing the potential for bias. Second, the study was limited to 24 h postoperatively, and long-term effects of sciatic and saphenous nerve blocks after ankle fracture surgery were not evaluated. Third, due to the study design involving patient blinding, the direct assessment of block success in patients could not be performed. Although ultrasound guidance was employed, the potential impact of the unintended spread of a small amount of local anaesthetic on analgesic outcomes cannot be entirely excluded. Fourth, although this study was a prospective randomised trial, the trial registration was completed retrospectively. However, this study was conducted in accordance with the IRB-approved protocol, and there were no protocol violations or changes to the prespecified outcomes. Further studies with a focus on additional postoperative outcomes, including nausea, vomiting, or agitation, might be needed to evaluate the comprehensive value of sciatic and saphenous nerve blocks for ankle fracture surgery.

## 5. Conclusions

In conclusion, ultrasound-guided sciatic and saphenous nerve blocks significantly reduced the immediate postoperative NRS scores and the total dose of remifentanil under qNOX-guided general anaesthesia during ankle fracture surgery. Regional analgesic techniques might be a good option for improving postoperative analgesia and reducing the use of opioids during the perioperative and immediate postoperative periods in patients undergoing painful ankle fracture surgery.

## Figures and Tables

**Table 1:** Patients’ characteristics.

	Nerve Block Group (*n* = 30)	Control Group (*n* = 30)	*p*-Value
Age, years (median)	49.1 ± 16.1 (54)	44.5 ± 14.6 (46)	0.173
Male	11 (36.7)	18 (60.0)	0.071
Body mass index, kg/m^2^	23.7 ± 3.4	25.3 ± 3.9	0.130
ASA score (I/II/III)	(11/14/5)	(10/18/2)	0.163
Hypertension	6 (20.0)	2 (6.8)	0.129
Diabetes	3 (10.0)	2 (6.7)	0.640
Operation site			0.121
Right	11 (36.7)	17 (56.7)	
Left	19 (63.3)	13 (43.3)	

Values are presented as the mean ± standard deviation, median (range), or number (%). ASA, American Society of Anaesthesiologists.

**Table 2:** Anaesthesia profiles.

	Nerve Block Group (*n* = 30)	Control Group (*n* = 30)	*p*-Value
Total anaesthesia time (min)	136.2 ± 37.6	141.9 ± 54.7	0.640
Total remifentanil dose (mcg)	1148.1 ± 391.8	1487.4 ± 798.8	0.041
Total propofol dose (mg)	1047.6 ± 391.8	1279.1 ± 629.2	0.084

Values are presented as the mean ± standard deviation.

**Table 3:** Postoperative profiles.

	Nerve Block Group (*n* = 30)	Control Group (*n* = 30)	*p* Value
NRS at PACU	2 (1–5)	7 (5–8)	<0.001
NRS at 6 h	3 (2–5)	4 (3–5)	0.068
NRS at 24 h	2 (1–4)	2 (1–4)	0.740
Patient satisfaction scale	3 (2–4)	3 (2–4)	0.206
QoR40 at 24 h	169 (155–184)	175 (154–165)	0.324
Rescue analgesic use	5 (16.7)	3 (10.0)	0.706
Adverse events	1 (3.3)	2 (6.7)	>0.99
Hypotension	1 (3.3)	1 (3.3)	
Shivering	0 (0)	1 (3.3)	

Values are presented as the median (interquartile range) or number (%). NRS, numeric rating scale; PACU, post-anaesthesia care unit.

## Data Availability

The datasets used for the current study are available from the corresponding author on reasonable request.

## Abbreviations

The following abbreviations are used in this manuscript:

NB	Nerve Block
NRS	Numeric Rating Scale
PACU	Post-Anaesthesia Care Unit
PCA	Patient-Controlled Analgesia
POD	Postoperative Day
QoR-40K	Quality of Recovery-40 Korean
TIVA	Total Intravenous Anaesthesia
